# A Transformer-Based Neural Machine Translation Model for Arabic Dialects That Utilizes Subword Units

**DOI:** 10.3390/s21196509

**Published:** 2021-09-29

**Authors:** Laith H. Baniata, Isaac. K. E. Ampomah, Seyoung Park

**Affiliations:** 1 School of Computer Science and Engineering, Kyungpook National University, 80 Daehak-ro, Buk-gu, Daegu 41566, Korea; laith@knu.ac.kr; 2 Department of Computer Science, Durham University, Stockton Road, Durham DH1 3LE, UK; Isaac.k.ampomah@durham.ac.uk

**Keywords:** neural machine translation (NMT), transformer, Arabic dialects, modern standard Arabic, subword units, multi-head attention, shared vocabulary, self-attention

## Abstract

Languages that allow free word order, such as Arabic dialects, are of significant difficulty for neural machine translation (NMT) because of many scarce words and the inefficiency of NMT systems to translate these words. Unknown Word (UNK) tokens represent the out-of-vocabulary words for the reason that NMT systems run with vocabulary that has fixed size. Scarce words are encoded completely as sequences of subword pieces employing the Word-Piece Model. This research paper introduces the first Transformer-based neural machine translation model for Arabic vernaculars that employs subword units. The proposed solution is based on the Transformer model that has been presented lately. The use of subword units and shared vocabulary within the Arabic dialect (the source language) and modern standard Arabic (the target language) enhances the behavior of the multi-head attention sublayers for the encoder by obtaining the overall dependencies between words of input sentence for Arabic vernacular. Experiments are carried out from Levantine Arabic vernacular (LEV) to modern standard Arabic (MSA) and Maghrebi Arabic vernacular (MAG) to MSA, Gulf–MSA, Nile–MSA, Iraqi Arabic (IRQ) to MSA translation tasks. Extensive experiments confirm that the suggested model adequately addresses the unknown word issue and boosts the quality of translation from Arabic vernaculars to Modern standard Arabic (MSA).

## 1. Introduction

The area of Machine Translation (MT) is undergoing unbelievable development thanks to deep learning and artificial neural network models. Although a few years ago, machine translation research tried to produce a high-quality translation for the most popular and resourceful languages, today’s level of translation quality has increased the need and significance of low-resource languages and the solution of further and more interesting translation tasks [1]. In particular, even national language varieties such as Arabic dialects, which are practiced by large populations (450 million) in the Arab world, lands as a spoken verity of modern standard Arabic (MSA) and has been largely ignored by industry and research. At the moment, commercial machine translation services do not provide translation services for any Arabic vernaculars. Conventional translation systems that perform translation from Arabic dialects to MSA generate inconsistent outputs such as mixing lexical parts. These systems translate parts of the source sentence twice and do not produce high translation quality. Moreover, in Arabic, a linguistic phenomenon known as diglossia occurs, in which language speakers practice local vernaculars for informal environments and they practice modern standard Arabic language for formal contexts. For example, communities in Morocco use both “standard” Arabic and Maghrebi vernacular, depending on the context and situation. This Maghrebi vernacular reflects their own identity, history, lived experiences, and culture. Dialects by region are immense, such as Levantine, Maghrebi, Yemeni, Iraqi, Nile basin (Egypt and Sudan), and Gulf. Still, Arabic vernaculars also change even within individual Arabic-speaking countries. A further difficulty is mixing Arabic dialects and modern standard Arabic language together. To illustrate the importance of dealing with Arabic vernaculars, Ethnologue reported that Arabic has the 5th largest number of L1 speakers scattered all over 21 regional vernaculars. There are four types of machine translation: statistical machine translation (SMT), rule-based machine translation (RBMT), hybrid machine translation and neural machine translation (NMT).

Traditional methods such as statistical machine translation (SMT) require powerful computing devices. SMT is not suitable for managing the problem of word order, one of the Arabic vernacular’s main syntactic problems. To study the word order, we need to know where the verb, object and subject in the phrases are. According to the research studies, languages can be categorized as verb-object-subject VOS (Arabic), subject-object-verb SOV (Hindi language), subject-verb-object SVO (English), and other languages such as Arabic vernaculars that allow the free word order feature. The word order does not only convey any information about the subject and the object but possible different information (old and new). These profound differences pose a challenge to the statistical translation systems due to the fact that as sentences become lengthier, they do not just contain an object, verb, and a subject, but instead, the sentence will have a complex structure made up of several parts. In the case of Neural Machin Translation (NMT) systems, the encoder compresses the input sequence into a single vector representation as noted in the encoder-decoder structure, where the decoder uses this vector representation to produce the output sequence. However, this structure has the disadvantage that input sequence information is lost and the quality of translation declines when the input sentence is longer. Furthermore, the lack of standardized spelling for Arabic dialects presents a challenge in developing an NMT models for these vernaculars. The lack involves morphological dissimilarities which are apparent by using affixes and suffixes that are not used in MSA. Basically, for NMT systems training, we need large amounts of annotated data, which is not possible in languages with low resources such as Arabic vernaculars. Moreover, the quality of translation is decreasing alongside a decrease in the amount of the training data for low resource languages. 

In Arabic dialects, the translation of rare words is a clear problem. Typically, there are 30,000–50,000 words confined to the neural model’s vocabulary. Nonetheless, translation is an open-vocabulary problem, mainly in languages that use productive word-formation processes such as compounding and agglutination; models of translation require methods below word level. For instance, in word-level NMT systems, the translation for the out-of-vocabulary words was discussed via back-off to a dictionary lookup [2,3]. We note that these techniques usually make incorrect assumptions in reality. For instance, due to the differences in the morphological synthesis between Arabic vernacular and modern standard Arabic language, one-to-one connection between source words and target words is not constantly occurring. Furthermore, word-level NMT systems are ineffective in translating and generating unseen words. One of the approaches is to copy unknown words into the target text as done by [2] and [3]. It is a suitable strategy for names, but it requires transliteration and morphological changes, particularly when the characters are different. In the case of transformer model that was proposed newly [4], it has outperformed recurrent neural network (RNN)-based models [5,6,7] and convolutional neural network (CNN)-based models [8] on various translation tasks, drawing the attention of MT researchers. The Transformer model, which applies a self-attention approach to measure the strength of a relationship within two words in a sentence, has contributed to raising performance in MT and various natural languages processing tasks, for instance, semantic role labeling and the language modeling. The techniques to tackle the difficulties of Arabic vernaculars translation are under research and investigation. There has been no earlier research project that concentrated exclusively on developing a Transformer-based NMT model running from Arabic vernaculars to modern standard Arabic language at the level of subword units. 

A Transformer-based NMT model is presented in the current research, using subword units to perform translation tasks from various Arabic vernaculars to modern standard Arabic language. Moreover, this research study introduced and developed a Word-Piece model to create subword units for the Arabic dialects. Experiments showed that machine translation tasks, computed using Bilingual Evaluation Understudy (BLEU) metric and human evaluation metric, have been enhanced on the performance of Arabic vernaculars to modern standard Arabic language. Furthermore, we found that the proposed NMT subword model based on transformers achieves higher efficiency for the translation of scarce words in comparison with models that have a large vocabulary and back-off dictionaries. The model can produce new words that are not seen during training time. Moreover, the proposed Transformer-based NMT subword model achieved high translation accuracy per sequence for Arabic dialects. Additionally, the research investigated the impact of training the model with subword embeddings and with different dimensions. Moreover, this research study investigated the influence of utilizing subword units on the Arabic dialect’s translation quality. This research project investigated the impact of training the model with a different number of encoders and decoders and with a different number of attention heads in the self-attention (MHA) sub-layer in the decoder and encoder.

## 2. Related Work

Despite that machine translation research area has been investigated for several years and decades, the majority of research effort has focused on high-resource translation pairs, for instance, French–English and German–English which have many free parallel datasets. Nevertheless, most language pairs in the world do not have large parallel data. Research attention in these low-resource translation settings has been growing during the last five years. Translations from and to written language varieties are mainly based on phrase-based SMT systems, such as those for Croatian, Serbian and Slovenian [9], Hindi and Urdu [10], and Arabic vernaculars [11]. Pourdamghani et al. [12] developed an unsupervised deciphering design to translate similarly associated languages with no need for parallel training data. Costa-jussà [13] showed the comparison of the Catalan–Spanish language pairs amongst rule-based systems, phrase-based systems, and NMT systems. The performance of NMT is better and more reliable than other systems when an in-domain test set is applied. Experiments in the out-of-domain test dataset have shown that better performance was provided by the rule-based method from Spanish language to Catalan language and phrase-based method from Catalan language to Spanish language. In order to translate texts from Kurman to Sorani, Hassani [14] introduced and developed an Intralingual MT model. The model performed a word-to-word translation either direct or literal translation between Kurman and Sorani dialects. The outcomes have been estimated by native speaker evaluators. The experiments confirmed, according to human raters, that this strategy can produce significantly clear results. Experiments also revealed that this strategy could be regarded as a fundamental solution to the lack of corpus problem.

The first NMT system that was trained to translate among language varieties was presented by Costa-jussà et al. [15]. The authors utilized language variety pairs, European Portuguese and Brazilian Portuguese for experiments, as well as a corpus of subtitles for neural machine translation training. The authors gained an additional 0.9 BLEU points for translating from European Portuguese to Brazilian Portuguese compared to the SMT system trained on similar data and an additional 0.2 BLEU points when translating in reverse direction. The results show that the neural machine translation model offers more reliable translation in terms of BLEU scores and seven native speakers’ evaluation than the SMT model. Lakew et al. [16] investigated NMT training difficulties from English into special pairs of language varieties, analyzing parallel texts and low-resource situations, both labeled as well as unlabeled. The authors conducted experiments from English to two languages, European Brazilian Portuguese and European Canadian French and two standardized pairs, from Croatian language to Serbian and from Bahasa Indonesia to Malay. The researchers demonstrate that a significant BLEU score increases over basic models when translation into related languages is learned as a multilingual task with shared representations. 

The main focus of research for Arabic vernaculars has been on SMT and rule-based methods. PADIC is a multi-dialect-Arabic corpus that was introduced by Meftouh et al. [17]. The PADIC corpus includes MSA, Levantine vernaculars (Syrian and Palestinian) and Maghrebi vernaculars (Tunisian and Algerian). In comparison to many other approaches, diverse experiments were applied on different SMT models with all language pairs (vernaculars and standard Arabic). In changing the smoothing methods, the researchers examined the influence of the language model on MT by interpolating them with a larger one. The most reliable translation outcomes were obtained in Algerian vernacular, which is not remarkable because there is no closeness between the Algerian vernacular and the MSA; therefore, the SMT model during training could not capture the whole semantic and syntactic features of Algerian vernacular. It also was noticed that because of the closeness of the vernaculars, MT performances within Palestinian and Syrian were relatively high. As far as MSA is concerned, Palestinian vernacular has achieved the most reliable results of MT. Sadat et al. [18] presented an approach to do translation of the Tunisian social media vernacular into modern standard Arabic. This system depends on a bilingual lexicon that was designed for this translation task. A collection of syntactic mapping rules alongside a disambiguation phase is used to choose the most appropriate translation phrases, depending on language model for MSA. The translation system should be noted as word-based. By using a test dataset of 50 sentences of Tunisian vernacular, it achieves a BLEU score [19] of 14.32 (the reference was done by hand). Bakr et al. [20] proposed a comprehensive system for translating Egyptian vernacular phrases to enunciated versions of modern standard Arabic phrases. The authors applied the statistical method to tokenize and tag Arabic phrases. The technique for producing diacritics for the target phrases in MSA was explicitly selected based on important rules. The research was assessed using a dataset that contains 1000 Egyptian vernacular sentences where the training set is 800 and the test set is 200. The method obtained a performance of 88% when translating vernacular words to modern standard Arabic words and an accuracy of 78% when generating the words in their correct order.

The majority of the methods discussed earlier concentrated on SMT system and rule-based system. The rule-based translation method has a notable shortcoming: the development of the before-mentioned methods requires a significant quantity of time. It is essential to adjust the rules to raise the rule-based MT quality, which needs an exceptional degree of lingual understanding. The statistical methods require high computing devices and these methods are unable to manage one of the Arabic vernacular syntactic problems: the problem of word order. There have been relatively few publications in NMT discussing the translation of closely related languages. Multitasking is widely regarded as a highly effective technique for boosting the effectiveness of translation for Arabic vernaculars. A new study that investigates NMT for Arabic vernaculars was first introduced by Baniata et al. [21]. For translation from Arabic vernaculars to modern standard Arabic, the researchers presented a multi-task neural machine translation system. The suggested system is based upon multitask learning, where the language pairs share a single decoder and every source language has a separate encoder. The practical experiments demonstrate that by employing small amount of training dataset, the multitask NMT model can generate a correct MSA phrase and produce a translation with very good quality and learning the predictive information of various targets at the same time. Among many methods to translate Arabic dialects, one of the most significant is the incorporation of outer knowledge into the neural network models for Arabic dialects. Baniata et al. [22] proposed a Multitask NMT model that shares an encoder between two types of tasks; Arabic vernacular to modern standard Arabic translation task and POS task on segment level. Between translation tasks, the system shares two layers; shared layer and invariant layer. By alternatively training translation and POS tagging tasks, the proposed model may exploit distinctive knowledge and enhance the translation effectiveness from Arabic vernaculars to modern standard Arabic. Practical experiments involve translation tasks from Levantine Arabic to modern standard Arabic and from Maghrebi Arabic to modern standard Arabic. 

Nguyen et al. [23] created a lexical semantic framework for unique features of Korean text as an information database to develop a morphological analysis and word sense disambiguation system called Utagger. Moreover, the authors created a corpus for Korean–Vietnamese where they utilized the word segmentation algorithm RDRsegmenter for Vietnamese text and Utagger for Korean text. This research team was able to build a bidirectional Korean–Vietnamese NMT system, using the encoder-decoder approach with attention. These experimental findings showed that the usage of UTagger and RDRsegmenter in the Korean–Vietnamese NMT system might increase its performance, obtaining exceptional outcomes from Korean to Vietnamese with BLEU score of 27.79 and TER score of 58.77 and in reverse way a BLEU score of 25.44 and TER score of 58.72. Park et al. [24] proposed the first ancient Korean NMT system based on the use of a Transformer. The method improves translator performance by instantly generating a draft translation for different ancient documents that remain untranslated. Moreover, shared vocabulary and the entity restriction byte pair encoding is a new subword tokenization approach that was proposed by the authors recently. This approach depends on the textual characteristics of ancient Korean sentences. By using this proposed approach, the effectiveness of the traditional subword tokenizing approaches such as the byte pair encoding will rise by 5.25 BLEU points. Additionally, several decoding algorithms such as the ensemble models and n-grams blocking contribute an additional 2.89 BLEU points to the performance. Luo et al. [25] suggested an NMT model in which the network is trained sequentially on not closely related high resource language pairs, intermediate language pairs which is related and low resource language pairs. These parameters are transferred and tuned from one layer to another for initialization step in the same way. Thus, the hierarchical transfer learning design unites data amounts benefits of languages with large resources with grammatical propinquity benefits the related language. For data preprocessing, the researchers applied byte pair encoding and character level embedding, which completely address the issue of shortage of vocabulary (OOV). Experiments analyzing Uygur–Chinese and Turkish–English translations illustrate the suggested method’s superiority over the neutral machine translation model with parent–child framework. Few publications have been published on the subject of MT for Arabic vernaculars that employ subword units. 

Aqlan et al. [26] suggested employing a romanization method that turns Arabic texts into subword units. The authors analyzed the impact of this strategy on Neural MT performance in various segmentation settings and measure the findings to methods trained on modern standard Arabic. Additionally, the authors combine Romanized Arabic text as an input component for Arabic-sourced neural machine translation compared to well-known components, including lemma, POS tags, and morph characteristics. The experiments that were performed on Arabic–Chinese translation show that recommended methodologies address the unknown word issue and improve the translation quality for the Arabic source language. This work carries out further experiments with the NMT system and develops it on Chinese–Arabic translation. Prior to conducting the experiments, the researchers created a criteria for filtering the text in the parallel corpus to remove the noise. Included sentence patterns have been shown to improve the performance of MT, particularly SMT and RNN-based NMT [27,28,29]. Further, Strubell et al. [30] have enhanced a Transformer-based SRL design by adding dependency formations of phrases into self-attention, which is named linguistically-informed self-attention (LISA). In LISA, one of the attention heads of a multi-head self-attention system is trained with constraints based on dependency relations to attend to syntactic parents for each token. 

## 3. Background

Recently, the NMT has been offered as an exciting framework that has the possibility to overcome the shortcomings of the standard SMT methods. The strength of the NMT approaches is their capability in learning the mapping from the input text to the corresponding output text directly, in an end-to-end pattern. Neural models in the domain are not new, as Neco et al. [31] proposed an approach years ago. Other models [32,33] were introduced later, but Chao et al. [6] and Sutskever et al. [7] were the first to design a robust machine translation system. Peyman et al. [34] presented an encoder-decoder structure in which two RNNs are trained to maximize a target sequence’s conditional probability (possible translation). y = $y_{1}$, …, $y_{m}$, given a source sentence x = $x_{1}$, …, $x_{n}$. Sequentially, the input words are processed until the end of the input string is reached. The encoder reads the input sequence and turns it into fixed length representation. Every time in step t an input word is received; the hidden state is updated. Equation (1) illustrates this process:

(1) $$h_{t}=f\left(E_{x\;}\left[x_{t}\right],h_{t-1}\right)$$

where $h_{t}\in R^{d}$ is the hidden state (vector) at the time step t and $f\left(.\right)$ is a recurrent function such as the long short-term memory (LSTM) [35] or the gated recurrent unit (GRU). $f\left(.\right)$ is reasonable for updating the hidden state of the layer and other associated unit (if there are any, such as memory unit, etc). $E_{x}\in R^{\left|V_{x}\right|\times d}$ is an embedding matrix for the source symbols (d is the embedding size). The embedding matrix is a lookup table whose cells are treated as a network parameters and updated during training. The embedding (numerical vector) for the vth word in $v_{x}$ (vocabulary) resides in the vth row of the table. In the next step, the model undertakes processing for all words in the source sentence; $h_{n}$ is a summary of input sequence, referred to as context vector (c). Another RNN is initialized by c and seeks to produce a target translation. There is one word sampled from a target vocabulary $v_{y}$ at each step of the process. The decoder conditions the probability of picking a target word $y_{t}$ on the context vector, the last predicted target symbol, and the decoder’s sate. This can be expressed in Equation (2):

(2) $$y_{t}=g(E_{y}[y_{t-1}],S_{t},c)$$

$S_{t}=f\left(E_{y}\left[y_{t-1}\right],S_{t-1},c\right)$ where $S_{t}$ is the hidden state of the decoder. Since we compute the probability of choosing $y_{t}$ as the target word, g(.) should give a value in the range [0, 1]. The most common function for $g\left(.\right)$ is Softmax. The encoder and decoder RNNs are trained together to maximize the log probability of generating a target translation and are given an input sequence x, so the training standards can be defined as in Equation (3):

(3) $$\underset{\theta }{\max}\frac{1}{K}\overset{k}{\underset{k=1}{\sum }}\log(y_{k}|x_{k})$$

where θ signifies a set of network parameters and K denotes the training set’s size. As previously noted, the recurrent functions used in encoder-decoder models are not conventional mathematical functions.

## 4. The Proposed Transformer Based-NMT Model for Arabic Dialects That Utilizes Subword Units

Even while the translation is considered to be an open vocabulary issue, systems of NMT always work with word vocabularies that are fixed (names, numbers, dates, etc.). To address out-of-vocabulary (OOV) words, there are two broad approaches. One strategy is to try to copy and obtain scarce words from source language and place them in the target language (since most of the scarce words are numbers or names so the right translation is a copy), either through the use of an attention mechanism [2], external alignment approach [3], or through the use of a complex special purpose pointing framework [36]. An extra group of methods is the subword units such as the combined word/characters or more knowledgeable subwords [37]. Subword segmentation is fundamentally an algorithm used under the assumption that a word consists of a combination of several subwords. Even Arabic dialects and MSA are languages based on Arabic characters, and many words are made of subwords. Therefore, breaking into subword units through suitable subword detection can reduce the number of vocabularies and efficiently reduce sparsity. In addition to reducing sparsity, the most representative outcome of subword segmentation is a useful coping with unknown (UNK) tokens. The majority of the deep learning NLP algorithms, including Natural Language Generation (NLG), take sentences as inputs simply as word sequences. So, when UNK appears, the probability of the language model in the future is very ruined. Therefore, it is difficult to encode or generate suitable sentences. Especially for sentence generation such asautomatic machine translation, it is more difficult because it predicts the next word based on the previous word. However, through subword units’ utilization, it is possible to create a combination of known tokens by dividing UNKs such as new words or typo into units of subwords or characters. In this way, by eliminating UNK itself, you can completely cope with UNK and boosts the translation quality of Arabic vernaculars. Many word patterns were generated from the same origin and root in Arabic vernaculars and modern standard Arabic, fragmenting the data and generating scattered data. 

**Figure 1:**
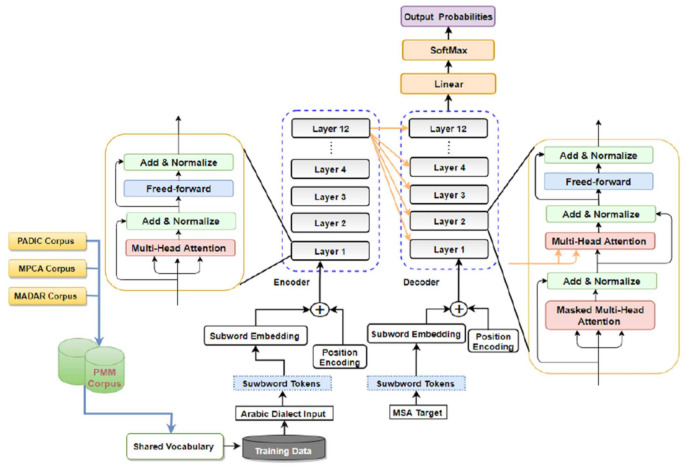
The Architecture of Transformer Based-Neural Machine Translation Subword Model for Arabic dialects.

Various research papers introduced statistical segmentation for the Arabic language in order to divide words down into their morphemes, which are the smallest meaningful unit in the language. This subtask is a fundamental part of a variety of natural language processing applications. For example, machine translation (MT) is powerful for the representation of the input and needs consistency across test and train data. Thus, by segmenting Arabic vernaculars and MSA words into subword units, the affixes and suffixes that are attached to the words are separated and the proposed model will capture more semantic and syntactic features of the input source sentence and produce a high-quality MSA sentence. This research paper developed an Arabic dialects Transformer-based NMT model that utilizes AD and MSA Subwords units to translate from different Arabic vernaculars to MSA. We created the model depend on the Transformer model introduced recently by Vaswani et al. [4]. For the proposed Transformer-Based NMT Subword model, as illustrated in Figure 1, both the decoder and encoder consist of a stack of 12 layers. Every layer has two different sub-layers: multi-head attention sub-layer and position wise feed forward sublayer (FFN). The encoder and the decoder in the suggested Transformer NMT model architecture for Arabic dialects make use of an attention model and feed-forward net to create sequences of changeable lengths without the need to use the RNN unit or CNN unit. The operation of attention across the various layers is based on multi-head attention (see Section 4.1). An input sequence of symbol representations (source sentence) $X=\left(x_{1}\;,x_{2},\ldots ,x_{nenc}\right)T$ is mapped to an intermediate vector. Next, the decoder creates an output sequence (target sentence) $Y=\left(y_{1}\;,y_{2},\ldots ,y_{ndec}\right)T$, given the intermediate vector. Because the transformer architecture does not contain convolutional or recurrent structure, it encodes positional word information as sinusoidal positional encodings:

(4) $$P{(}_{pos\;,2i})=\sin\left(pos/10000^{2i/d}\right)$$

(5) $$P{(}_{pos\;,2i+1})=\cos\left(pos/10000^{2i/d}\right)$$

where pos is position, i is considered to be the dimension, and d is the dimension of the intermediate representation. At the first layer of both encoder and the decoder, the positional encodings computed by Equations (4) and (5) are summed to the input embeddings. The encoder subnetwork comprises a stack of L similar layers so that *L* is set to different numbers 12, 8, and 4.

Every encoding layer has two layers: a multi-head attention sub-layer and position wise feed forward sub-layer. To ease training and improve performance, residual connection mechanism [38] and a layer normalization unit (LayerNorm) [39] are employed around each sublayer. Formally, the outcome of every layer $l\;(H_{e}^{l})$ is calculated as below:

(6) $$S_{e}^{l}=LayerNorm\left(MHA\left(H_{e}^{l-1},\;H_{e}^{l-1},H_{e}^{l-1}\right)+H_{e}^{l-1}\right),$$

(7) $$H_{e}^{l}=LayerNorm\left(FFN\left(S_{e}^{l}\right)+S_{e}^{l}\right),$$

where $S_{e}^{l}$ is considered to be the output from multi-head attention sublayer calculated based upon source sentence representation of previous encoding layer $\left(l-1\right)$. Moreover, the decoder consists of a stack of L similar layers in which *L* is set to different numbers 12, 8, and 4. Unlike the encoder, every layer in the decoder consists of three sublayers, a multi-head attention sublayer and a position wise feed forward sublayer. However, the encoder decoder multi-head attention sublayer is placed between them. The (encoder-decoder) multi-head attention sublayer is utilized to perform attention calculations for the output of encoder $H_{e}^{L}$ particularly, output of every decoding layer $l\;(H_{d}^{l}$) is computed as:

(8) $$S_{d}^{l}=LayerNorm\left(MHA\left(H_{d}^{l-1},H_{d}^{l-1},H_{d}^{l-1}\right)+H_{d}^{l-1}\right),$$

(9) $$E_{d}^{l}=LayerNorm\left(MHA\left(S_{d}^{l},H_{e}^{L},H_{e}^{L}\right)+S_{d}^{l}\right),$$

(10) $$H_{d}^{l}=LayerNorm\left(FFN\left(E_{d}^{l}\right)+E_{d}^{l}\right)$$

where $S_{d}^{l}$ is considered to be the output of multi-head attention sub-layer computed from target representation from previous decoder layer $\left(l-1\right)$. $E_{d}^{l}$ is considered to be the output of the encoder decoder MHA sub-layer generated based upon $S_{d}^{l}$ and $H_{e}^{L}$. The top-level layer output ($H_{d}^{L}$) of the decoder is used by a linear transformation layer to generate the target sequence. Specifically, the linear transformation layer via Softmax activation computes probability distribution of the output for target vocabulary.

### 4.1. Multi-Head Attention (MHA)

**Figure 2:**
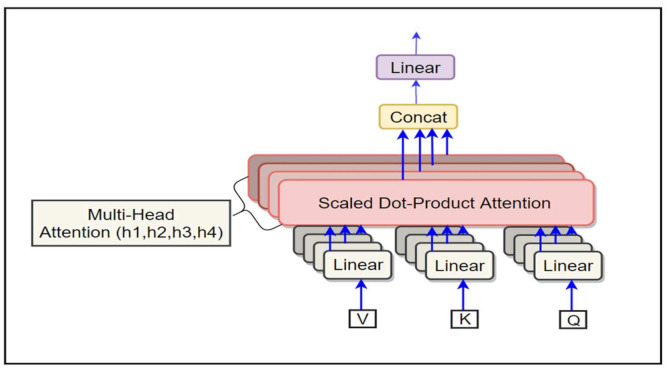
The Multi-Head Attention consist of several attention layers running in parallel.

A neural attention mechanism is an essential feature of the seq2seq structure, which is used to solve a variety of sequence generating challenges, including document summarization [40] and NMT. The Transformer Based-NMT subword model perform the scale dot product attention function as shown in Figure 2. This takes three vectors as inputs, the queries Q, values V and keys K. It outlines the provided query and key–value pairs to an output weighted sum of the values. The weights show the association among every query and key. An attention is illustrated below:

(11) $$Attention\left(Q,K,V\right)=softmax\left(\alpha \right)V$$

(12) $$\alpha =score\left(Q,K\right)$$

(13) $$score\left(Q,K\right)=\frac{Q\times K^{T}}{\sqrt{d_{k}}}$$

where $k\in R^{J\times d_{k}}$ is the key, $V\in R^{J\times d_{v}}$ is the value $Q\in R^{Z\times d_{k}}$ is a query. Z and J are considered to be the lengths of sequences expressed by Q and K, respectively. $d_{k}$ and $d_{v}$ are considered to be the dimension of value and key vectors, respectively. The query dimension is expressed by $d_{k}$ to perform the dot product calculation. 

The division of $Q\times K^{T}$ by $\sqrt{d_{k}}$ is performed to measure the output of the product operation so maintaining the calculation Vaswani et al. [4]. The overall attention weight distribution is obtained by applying the $softmax\left(.\right)$ operation to the attention score $\alpha \in R^{Z\times J}$. For better performance, the transformer architecture uses MHA, which comprises $N_{h}$ (number of head attentions) measured dot product attention operations. Provided the Q, K, and V, multi-head attention computation is shown below:

(14) $$MHA\left(Q,K,V\right)=O,$$

(15) $$O=HW_{o},$$

(16) $$H=concat\left(head_{1},head_{2},\ldots \ldots ,head_{N_{h}}\right)$$

(17) $$head_{h}=Attention\left(QW_{h}^{Q},KW_{h}^{k},VW_{h}^{v}\right)$$

where $QW_{h}^{Q},KW_{h}^{k}$ and $VW_{h}^{v}$ are projections of query, key and value vectors for hth head, respectively. These projections are made with metrics $W_{h}^{Q}\in R^{d_{model}\times d_{k}}$, $W_{h}^{k}\in R^{d_{model}\times d_{k}}$, $W_{h}^{v}\in R^{d_{model}\times d_{v}}$. The inputs to the MHA(.) are $K\in R^{J\times d_{model}}$, $V\in R^{J\times d_{model}}$ and $Q\in R^{Z\times d_{model}}$. $head_{h}\in R^{J\times d_{v}}$ is the output of measured dot product calculation for the hth head. The $N_{h}$ measured dot product operation are united by using the concatenation function $concat\left(.\right)$ to generate $H\in R^{Z\times \left(N_{h}.d_{v}\right)}$. Eventually, the output $O\in R^{Z\times d_{model}}$ is produced from the projections of H utilizing the weight matrix $W_{o}\in R^{(N_{h}.d_{v)\times d_{model}}}$. The MHA contains the same number of parameters as vanilla attention if

(18) $$d_{k}=d_{v}=\frac{d_{model}}{N_{h}}$$

### 4.2. Segmentation Approach: Wordpiece Model

The subword units are the best approach for handling the problems and challenges of Arabic dialects. This study uses the WordPiece Model (WPM) implementation, which was originally used by Google to tackle a Japanese–Korean segmentation challenge [41]. This method is entirely data-driven and guarantees that any possible Arabic dialect sequences are segmented in a deterministic way. It is similar to the approach used in Neural Machine Translation [37] to address rare words. To begin the process of the random words, we divide these words to word pieces using a trained wordpiece method. Prior to training the model, accurate word boundary symbols are added to ensure that original word sequence is extracted without ambiguity of word piece sequence. At the time of decoding, this model produces the wordpiece sequence, where this wordpiece sequence is reshaped to an identical word sequence. The example below illustrates the sequence of the word and equal word piece sequence for a sentence in Levantine Arabic vernacular:

Word: **“** وين فيي اركب عباص مخرج المدينة**”**
Word Translation: “Where can I take a bus to the city exit”
Wordpieces: **“** _ وين_ في ي_ اركب_ ع باص_ مخرج_ المدينة **”**

As illustrated in the above example: The Arabic word in LEV dialect **“**فيي**” “** I can **”** is decomposed into two-word pieces **“** في **” “**particle that derives a preposition from فيي **”** and **“**_ ي,**” “** particle that derives a suffix from فيي **”** while the word **“** عباص **” “** a bus **”** is decomposed into word pieces **“** ع **” “** particle that derives an affix from عباص **”** and **“**_ باص **” “**particle that derives a noun from عباص **”**. The remaining of the words are maintained as single word pieces. Wordpiece design is constructed by employing the data driven method which maximizes the language model probability for the training data given a word description. In the availability of parallel corpus for training and set of tokens R, the optimization challenge is by choosing R word pieces in a way where the final corpus contains the fewest word pieces when segmented by the word piece method. A unique token is utilized at the beginning of words rather than two ends. Additionally, the number of primary characters is decreased to a changeable number based upon the data. Moreover, the remaining characters are mapped to a particular unknown alphabet to avoid connecting word piece vocabulary with divided characters. 

We noticed that when exploiting a vocabulary within 100,000-to-24,000 word pieces, it leads to a high BLEU score and fast decoding quality for all language pairs that were evaluated. It is advantageous in translation to copy scarce names or numbers from source language to target language in a direct way. We utilized a shared word piece approach for the source language (Arabic dialect) and the target language (modern standard Arabic) to facilitate this type of direct copying. When this strategy is applied, the same string is segmented precisely in the same way in the source and target sentences, which makes it more straightforward for the model to copy these tokens. Word pieces accomplish stability between words’ efficiency and alphabets’ flexibility. The motivation behind can be summarized in two points. The first point illustrates that the processing for the source language (Arabic vernaculars) and the target language (MSA) is done by exploiting the shared vocabulary approach. The encoder of the proposed model shares the same vocabulary with the decoder. By applying the shared vocabulary approach between decoder and encoder, the proposed model substitutes the words in input sentence with translation words in target language. The second point, as illustrated by Lample et al. [42], a shared vocabulary enhances the alignment of embedding vectors. We also notice that when we use wordpieces, our suggested model achieves superior overall BLEU scores, which is most likely due to the proposed model’s ability to deal with an effectively unlimited vocabulary without simply relying on characters. The latter would require more computation and greatly increase average lengths for output and input sequences. 

## 5. Experimental Results

Multiple experiments were conducted to evaluate the proposed Transformer-based NMT subword system on a variety of translation tasks. The introduced model is evaluated on the basis of its ability to translate from Arabic vernaculars to modern standard Arabic. Practical experiments were carried out with five different dialects of Arabic: Levantine, Nile Basin, Maghrebi, Gulf and Iraqi. Levantine Arabic is an Arabic dialect spoken widely in Jordan, Syria, Lebanon and Palestine. The Maghrebi variety is commonly practiced in Algeria, Morocco, Tunisia, Libya. Arabic in Nile Basin is a popularly spoken dialect used in Egypt, Sudan and South Sudan. Gulf Arabic is a spoken dialect commonly spoken in KSA, UAE, Qatar, Oman, Kuwait and Bahrain. Iraqi Arabic is a dialect spoken in Iraq. For the language of low resources, the proposed Transformer-Based NMT subword model will be applied.

### 5.1. Data

**Figure 3:**
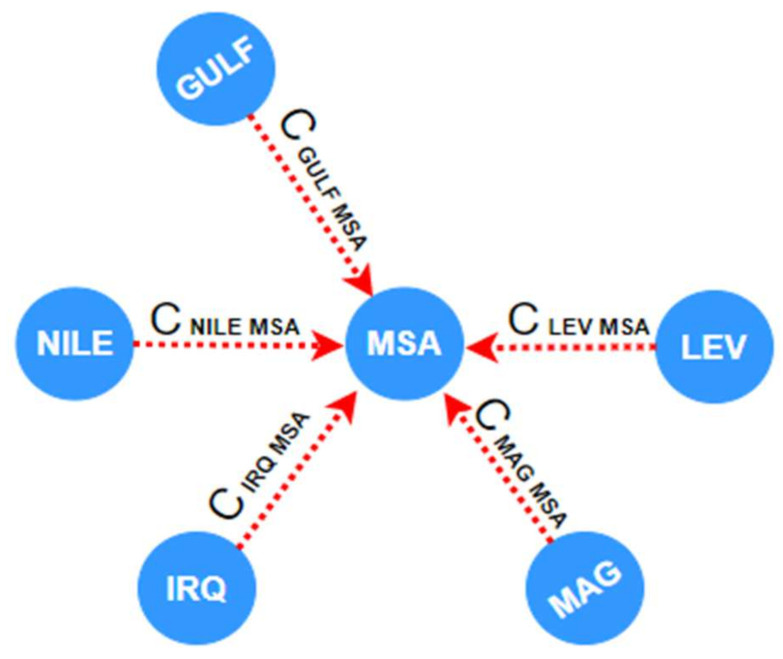
Translation graph: Arabic dialects (nodes), Parallel Corpora (dotted edges).

For the translation tasks, we grouped the Maghrebi vernaculars (Moroccan vernacular, Algerian vernacular, Tunisian vernacular and Libyan vernacular) unitedly from PADIC corpus [17], MPCA corpus [43] and MADAR corpus [44] into a single corpus, we will name it PMM-MAG. The Levantine vernaculars (Jordanian vernacular, Syrian vernacular, Lebanese vernacular and Palestinian vernacular), which are grouped collectively from PADIC corpus, MPCA corpus and MADAR corpus are grouped into a single corpus, and we will name it PMM-LEV. Furthermore, we concatenated Nile Basin Dialects (Egyptian vernacular, Sudanese vernacular) from MADAR corpus and the Gulf Dialects (Saudi Dialect, Omani Dialects and Qatari Dialect) are concatenated together from the same corpus. Moreover, we used the MADAR Corpus for the translation task of the Iraqi dialect. Figure 3 presents an example translation graph with nodes and dotted edges. We will use this graph as our running example. The Transformer NMT subword system was trained on 36,850 sentence pairs for Levantine vernacular, 54,736 sentence pairs for Maghrebi vernacular, 18,000 sentence pairs for Nile Basin Dialect, 18,000 sentence pairs for Gulf vernacular and 5000 sentence pairs for Iraqi vernacular. Textual information was gathered from many resources such as television episodes, films and social media. Regarding the test dataset, the proposed system was tested on 3000 sentence pairs for Levantine vernaculars, 3000 sentence pairs for Maghrebi vernacular, 2000 sentence pairs for Nile Basin vernacular, 2000 sentence pairs for Gulf vernacular, and 1000 sentence pairs for Iraqi vernacular. Moreover, the suggested system was trained with 13,805 sentence pairs for Levantine vernacular and it was trained on 17,736 sentence pairs for Maghrebi vernacular from the same corpus that was utilized by Baniata et al. [22] and tested on 2000 sentence pairs for Levantine vernacular and 2000 sentence pairs for Maghrebi vernacular.

The parallel corpus for each Arabic dialect was divided into two parts: 80% for training and 20% for testing. Additionally, each Arabic dialect’s test set was drawn from the same domain. The corpora employed in this study contain unprocessed information that may affect the proposed model’s performance. Therefore, all categories of Arabic vernaculars and modern standard Arabic sentences have undergone pre-processing. Hashtags, punctuation, non-Arabic characters and diacritics were excluded in Arabic vernaculars and modern standard Arabic. Additionally, the orthographic normalization process was applied. As an example, the characters إأٱآ were converted to the ا alphabet. Stop word removal or stemming have not been applied. The modern standard Arabic (MSA) includes various tokens found in Arabic dialects (AD), and the AD Sentences are shorter than those found in MSA. Three cross-validation strategies are commonly applied to determine a predictor’s predicted success rate: jackknife test approach, K-fold cross validation and independent dataset test. Among these procedures, the jackknife test is considered to be the least arbitrary and the most thematic, and as a result, it is popularly known and regularly chosen by researchers in order to evaluate the quality of different predictors. However, this strategy consumes the time and consumes the source because its estimated standard error tends to be slightly larger than other methods. Moreover, the jackknife test performs poorly when the estimator is not sufficiently smooth. Accordingly, K-fold cross-validation is applied in this paper, which sets K to 2 to create a train/ test split to assess the proposed Transformer-Based NMT Subword model. To avoid model overfitting, we used the Early stopping option where the patience parameter is set to 3 epochs and the model checkpoint is used to save the best weights for the evaluation of the proposed model.

### 5.2. Model Setup

The proposed model was developed using Python, Keras and TensorFlow. The experiments on the LEV–MSA, MAG–MSA, GULF–MSA, Nile–MSA and IRQ–MSA translation tasks are conducted based on these basic and advanced configurations where the subword embedding dimension has the three values which are 1024, 512 and 256, hidden state has two values which are 1024 and 512 and the attention heads are set to two values which are 8 and 4. The position-wise FFN has a filter of dimensions 512 and 1024. The proposed Arabic dialect Transformer-based NMT Subword model trained on the LEV–MSA, MAG–MSA, GULF–MSA, Nile–MSA and IRQ–MSA translation tasks consists of a 12, 8 and 4-layer encoder subnetwork. Moreover, it consists of a 12, 8 and 4-decoder subnetwork.

### 5.3. Traning and Inference

For the translation tasks LEV–MSA, MAG–MSA, GULF–MSA, Nile–MSA, and IRQ–MSA, the proposed model is trained for 13k iterations with batch size 2048 tokens and the maximum sequence length is set to 100 subword tokens. Moreover, the maximum subword tokens length is set to 150 subword tokens. The optimizer employed to train the model in this research study is Adam optimizer [45] with (β1 = 0.9, β2 = 0.98, ϵ = 1 × 10^−9^). Further, the number of epochs is set to 6 for all translation tasks. By following So et al. [46], a single cosine cycle with a warm up is applied for the learning rate schedule algorithm. The target sentences are produced by using beam search during the inference stage. For the LEV–MSA, MAG–MSA, GULF–MSA, Nile–MSA, IRQ–MSA, LEV–MSA (Baniata et al. [22] Corpus) and MAG–MSA (Baniata et al. [22] Corpus) translation tasks, beam size of 6 and length penalty of 1.1 are applied. The study used a shred vocabulary for the source language and target language. This research study employed 21,000-subword vocabularies for Levantine vernacular (LEV)—MSA translation task, 21,000 subword vocabularies for Maghrebi vernacular (MAG)–MSA translation task, 21,000 subword vocabularies for the Nile Basin Arabic (NB)—MSA translation task, 21,000 subword vocabularies for the Gulf Arabic (Gulf)—MSA translation task and 9235 subword vocabularies for the Iraqi Arabic (IRQ)—MSA translation task. Twenty-nine thousand five hundred subword vocabularies are employed for the corpus applied by Baniata et al. [24] on the Levantine Arabic (LEV)–MSA translation task and on the Maghrebi vernacular (MAG)–MSA translation task. Relu dropout value and attention dropout value are 0.1. The suggested model proved to be very fast and required 268 s per epoch for MAG–MSA task (Baniata et al. [22] Corpus), 419 s per epoch for LEV–MSA task (Baniata et al. [22] Corpus), 251 s per epoch for the MAG–MSA task, 216 s per epoch for Nile–MSA task, 254 s per epoch for LEV–MSA task, 231 s per epoch for Gulf–MSA task, 152 s per epoch for IRQ–MSA task. For each translation task, the proposed model is trained to minimize cross-entropy loss.

### 5.4. Results

#### 5.4.1. Automatic Metric

Many practical experiments were carried out with the proposed Transformer-based NMT model through exploiting subword units and shared vocabulary for Arabic vernaculars and MSA. The proposed Transformer-based subword model was experimented with different subword embeddings to find the most efficient subword embedding dimension of the proposed model. Moreover, the proposed model was trained with different number of encoders and decoders and with different number of heads in multi-head attention sublayer to find the most efficient number of attention heads for the proposed model. The translation quality is reported based on the sacreBLEU. SacreBLEU is a standard BLEU [19] implementation that manages WMT datasets, creates scores on detokenized outputs and reports a string encapsulating BLEU parameter, helping the generation of sharable, comparable BLEU scores. This section shows the performance evaluation of the suggested transformer-based NMT subword model for Arabic dialects on five Arabic dialects translation tasks. The findings of the LEV–MSA translation task are summarized in Table 1. For the MAG–MSA, Nile–MSA, Gulf–MSA and Iraqi–MSA translation tasks, Table 2, Table 3, Table 4 and Table 5 illustrate the results. Table 1 shows Transformer-Based NMT subword model results with different settings on the test dataset for the LEV–MSA translation task. The results in Table 1 show the effectiveness of the proposed transformer machine translation subword model. As illustrated from Table 1, the suggested model achieved an outstanding 63.71 BLUE score where the number of encoders’ layer and decoders’ layer value is 12, number of attention heads is 4 and the subword embedding value is 512. The findings are obvious as a result of the close connection between Levantine vernacular and modern standard Arabic, and the fact that both languages share a large number of vocabularies. It can be noticed from Table 1 that the experiments’ settings with low dimensions of subword embeddings achieved better BLUE score results in comparison to experiments with a high dimension of subword embeddings.

Table 2 illustrates the findings of the suggested model on test dataset for the MAG–MSA translation task. We observed that the proposed Transformer-based NMT subword model, as shown and highlighted with bold text in Table 2 is able to translate the Maghrebi Arabic sentences to MSA with a 65.66 BLEU score. It is clear that Maghrebi vernacular is a combination of many diverse languages such as the Berber language, African Romance, old Arabic expressions, Turkish language, Spanish, Italian and Niger Congo languages, as well as some new vocabularies borrowed from French and English. Therefore, the proposed Transformer-based NMT subword system was able to capture the semantic and syntactic features of Maghrebi dialect and improved the translation performance on the (MAG)–MSA translation task despite that the Maghrebi dialect is not close to the MSA in terms of expressions and vocabularies. Conventional NMT models [21,22] were not able to produce a high translation quality for the Maghrebi vernacular because Maghrebi dialect has expressions from many other languages. By utilizing subword units as an input to the encoder, there will be a sharing of information between the subwords forms and words forms and the model will generate MSA sentences with high quality.

Table 3 reveals the proposed model results with diverse settings on the test dataset for the Nile–MSA translation task. As seen from Table 3, the proposed system achieved a 48.19 BLUE score and the model was able to translate the Egyptian and Sudanese sentences to MSA correctly. Table 4 and Table 5 present satisfied results on Gulf–MSA, Iraqi–MSA translation tasks, respectively. The proposed model proved to produce high translation quality of Arabic Gulf sentences with a 47.26 BLEU score and 56.50 BLEU score on the Iraqi–MSA translation task. Furthermore, the model was applied on Maghrebi–MSA, Levantine–MSA parallel Corpora used by Baniata et al. [22]. It can be shown from Table 6 and Table 7 that the proposed Transformer-based NMT subword system has achieved 57.85 and 57.92 BLUE scores on Maghrebi–MSA, Levantine–MSA translation tasks, respectively. Therefore, it can be summarized as illustrated in Table 6, Table 7 and Table 8 that the proposed Transformer-Based NMT model that utilizes Arabic dialects subword units outperforms the multitask NMT system with part of speech tags that was proposed by Baniata et al. [22] on Maghrebi–MSA, Levantine–MSA translation tasks. These results were an indication of the effectiveness of utilizing subwords units and the usage of the shared vocabulary between Arabic dialect and MSA in the proposed model. Exploiting subwords’ units of Arabic dialects (Levantine, Maghrebi, Nile Basin, Gulf and Iraqi) as an extra feature in the Transformer-based machine translation model is advantageous for word order problem and it generated a high quality MSA sentences. By using subword units and Self-attention (multi-Head attention), the proposed model could better represent and obtain more semantic features from AD source language and solve the grammatical problem for AD: the word ordering issue.

#### 5.4.2. Human Evaluation

The human evaluation experiments confirm the results that were obtained by the automatic evaluation. The pilot rating experiments were selected [15]. Participants were requested to evaluate the translations on a 1 to 7 Likert metric. We evaluated the translation quality for LEV–MSA, MAG–MSA, Nile–MSA, Gulf–MSA and Iraqi–MSA tasks asking seven speakers who know modern standard Arabic and understand every Arabic vernacular to evaluate sentences generated from the proposed transformer NMT subword model. We offered to the speakers a segment in LEV, MAG, Gulf, Nile and Iraqi and one translation in MSA for each Arabic dialect. We selected at random 100 segments and divided them into five subsets of twenty segments each. We give every annotator a subset and request them to evaluate the translations considering adequacy and fluency applying Likert metric from 1 to 7. The average results that were produced through every model using pilot rating experiments are illustrated in Table 9 and Table 10. The average results pointed out that native speakers have positive and real judgment regarding the translations generated through the proposed Transformer-Based NMT subword model. The average score on the LEV–MSA translation task captured by multitask NMT part of speech tags system that was proposed by Baniata et al. [22] was 5.9. Furthermore, the average score on the MAG–MSA translation task captured through multitask NMT with part of speech tags system was 4.4. The average score on LEV–MSA translation task captured through the Transformer-Based NMT subword model is 6.0 and the average score obtained for the MAG–MSA is 6.2. Moreover, the average score for Gulf–MSA, Nile–MSA and Iraqi–MSA translation tasks obtained by the proposed model is 5.85, 5.8, 6.10, respectively. The findings of pilot rating experiments give confirmation that the Transformer-Based NMT subword model (T-NMT-Subword) generates better translation quality than the multitask NMT with part of speech tags system for all translation tasks.

## 6. Analysis

This analysis clarifies the positive effect of utilizing subword units on Arabic vernaculars to modern standard Arabic translation task efficiency. Table 11 shows sample translations from the Transformer-based NMT subword model on the LEV–MSA, MAG–MSA, Gulf–MSA, Nile–MSA, and Iraqi–MSA translation tasks. Due to the lack of standardization for the Arabic vernaculars, Conventional NMT methods for Arabic dialects are incapable of translating parts of input source sentences. Affixes and clitics are not obtained and captured effectively without the need to utilize the Subword units and multi-head attention. In Table 11, the proposed Transformer-Based NMT subword model translated 100% of Maghrebi vernacular sentences correctly. For Levantine Arabic, the proposed model translated 99% of whole Levantine Arabic phrases properly with the exact meaning regardless the word بعض الاصدقاء “some friends” in the generated sentence is not the same word اصدقائي “my friends” in the reference sentence, but it gives the same meaning. For the Gulf Arabic, the proposed model was able to translate 95% of the Gulf Arabic sentence fluently except the words مثل ما تشوف “as you see “, the proposed model could not translate them well and translated it to لن يكون “it will not be “rather than انت ترى “you see”. Furthermore, the Transformer-based NMT subword model achieves the overall best translation performance for Nile–Arabic and Iraqi–Arabic sentences. The translation quality of the suggested Transformer-Based NMT subword system for Arabic vernaculars has enhanced in comparison to Multitask NMT system (with POS tags) that was recently suggested by Baniata et al. [22] that applied experiments on the same corpus as seen in Table 12. The proposed Transformer-Based NMT subword model translated the source sentences of Maghrebi dialect and Levantine dialect to MSA fluently and with high translation quality without any translation mistakes. 

The influence of utilizing subword units and shared vocabulary between Arabic dialects and MSA and applying the self-attention mechanism on translation quality for Arabic vernaculars is significantly evident. The proposed Transformer-Based NMT Subword model is well-suited to handle the issue of free word ordering and create a right context and a correct order for the target language sentences, as illustrated in Table 11 and Table 12. Furthermore, the proposed system can obtain excellent translation effectiveness with various language pairs, as illustrated in Table 11 and Table 12 for MAG–MSA, LEV–MSA, Nile–MSA, Gulf–MSA and IRQ–MSA tasks. The proposed model was evaluated on the same parallel corpus that was applied by Baniata et al. [22] for Maghrebi Arabic and Levantine Arabic. Compared to the multitask NMT with part of speech tags system that was trained on the same parallel corpus, the suggested Transformer NMT subword system scored an effectiveness of 56.49 BLEU score for translating from Levantine Arabic vernacular to MSA and 57.06 BLEU score for translating from Maghrebi Arabic vernacular to MSA. The findings show that the proposed Transformer NMT subword model performs outstanding translation quality than the multitask NMT with part of speech (POS) tagging system by evaluating the systems using BLEU score and experiments of human assessment. 

**Figure 4:**
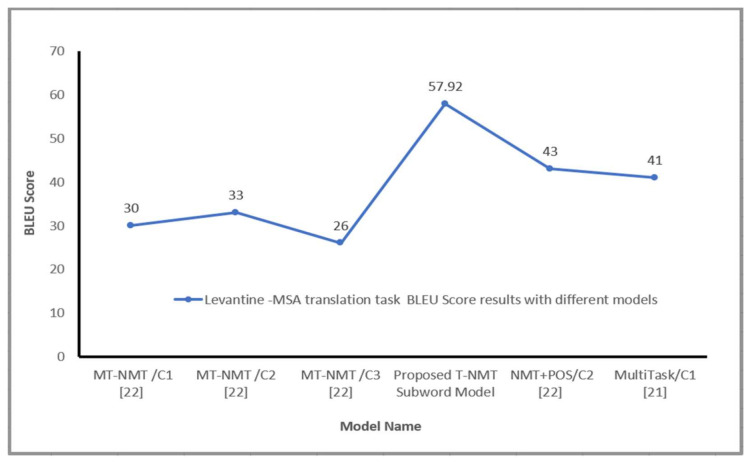
Levantine Arabic-MSA BLEU Score with different models, where C 1 is a random embedding, C 2 a pre-trained/Fast-text, C 3 a pre-trained/Polyglot.

**Figure 5:**
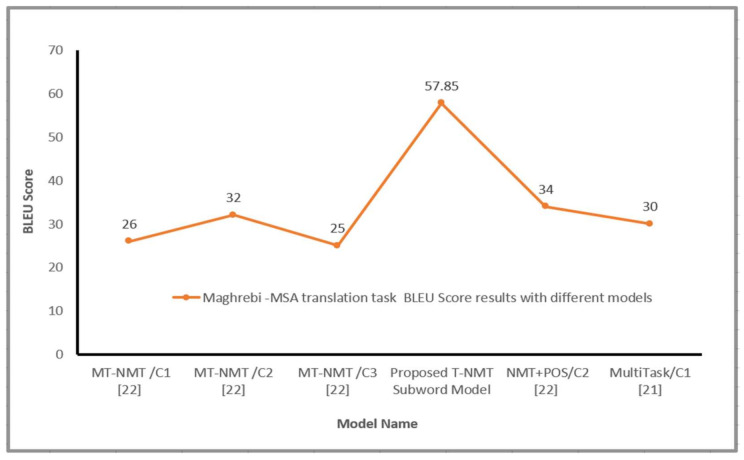
Maghrebi Arabic-MSA BLEU Score with different models, where C 1 is a random embedding, C 2 a pre-trained/Fast-text, C 3 a pre-trained/Polyglot.

The Transformer-based NMT subword model obtained remarkable results BLEU score for all Arabic dialects in comparison with various NMT systems, as shown in Figure 4 and Figure 5. It should be noted that the representation of source language learned via the proposed model significantly improves the translation effectiveness for language pairs. Generally, the suggested model is capable to produce fluent sentences in MSA language and transfer the information regarding the subject, object and verb for a free word order language such as Arabic vernacular. This section presents more analysis to know the effect of utilizing subword units on the performance of the proposed Transformer NMT subword system. This analysis includes (a) contribution of different numbers of encoder layers to the translation effectiveness of the proposed system (b) impact of translation quality regarding source sentence length, (c) impact of varying beam size concerning the effectiveness of the suggested model, (d) the impact of the encoder’s self-attention and, (e) quantitative analysis of the proposed model. These analyses are applied to the MAG–MSA because of the dataset’s size and the number of layers used to train the model. 

### 6.1. Impact of Hayperparameter n

Table 1, Table 2, Table 3, Table 4, Table 5, Table 6 and Table 7 show that performing the Transformer-Based NMT subword model across various source representations obtained from several encoding layers significantly enhances the performance of the proposed model for all Arabic vernaculars. n represent the number of encoding layers in the proposed Transformer-Based NMT subword model. This part studies the impact of varying the value n (using only the representations from top n encoding layers). The proposed Transformer based NMT subword model is trained with various values of **n** where **n** is set to 4, 8 and 12. Where n = 4 indicates a setup for a simple model, **n** = 8 indicates a configuration for a medium-sized model and n = 8 indicates a configuration for a large model. As seen from Table 2, for example, in the Maghrebi Arabic-MSA task (and in all translation tasks), there is (in most cases) a significant change in BLEU score as the value of n changes.

### 6.2. Length of Source Sentence

**Figure 6:**
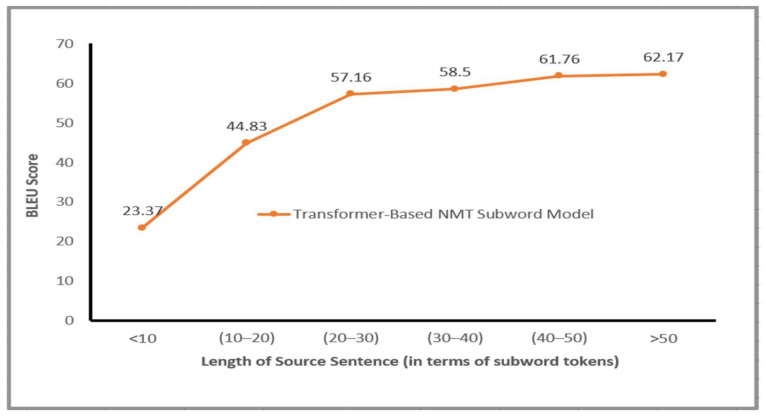
BLEU Score on MAG–MSA test dataset for the Transformer-NMT Subword model with respect to different source sentence length.

Obtaining contextual information and long-distance dependencies between the source sentence’s tokens can considerably increase the performance of longer sentences’ translation. As mentioned by (Luong et al. [47]) sentences that have the same lengths (number of source tokens) are collected together. The grouping is arranged by the lengths of source sentences (the number of subword tokens in every source sentence) over the MAG–MSA test set. We selected the MAG–MSA translation task to investigate the translation quality of long sentences because of the large size of the MAG–MSA corpus. The comparison in this research project is based upon these lengths: >50, 40–50, 30–40, 20–30, 10–20 and <10. Regarding every length interval, BLEU score metric is computed for the output of the suggested Transformer-Based NMT subword model. As can be noted in Figure 6, the proposed model performance improves while the input sentence lengths increase, particularly for the lengths between 40 and 50 subword tokens and for the lengths larger than 50 subword tokens with 61.76 and 62.17 BLEU scores, respectively. The proposed model, through the use of self-attention sublayers, is capable of modeling or obtaining contextual knowledge and dependencies within the tokens regardless of their distance or location within Arabic dialect sentence input. However, the performance of the suggested model decreased for very short sentences that have lengths smaller than 10 (in terms of the number of subword tokens). The performance increased for sentences with lengths larger than 50 (in terms of the number of subword tokens). Moreover, the proposed model performed inadequately on few numbers of short sentences with length less than 10 subword tokens with the lowest BLEU score (23.37). This occurs because these very short AD sequences contain only subword tokens (suffixes, affixes and morphemes) and they are cannot be aligned to the corresponding words in the target language. Overall, the performance of the proposed Transformer-Based NMT subword model obtained across the different groups motivates the hypothesis that employing subword units and using shared vocabulary between source language (Arabic dialect) and target language (MSA) enhances the encoder’s self-attention sublayers’ effectiveness in effectively capturing the global dependencies between words in the input sentences.

### 6.3. Beam Size Evaluation 

A comparison is conducted for the performance of the proposed model by modifying the beam size. The experiments are carried out based on the Transformer NMT subword model for Arabic vernaculars. We employed Wordpiece model for subword tokenization. Beam size has a large impact on the decoding speed (as words per second) and translation quality (in BLEU). Table 13 shows the outcomes of the experiments on the MAG–MSA translation task and results show that the optimum performance (in BLEU) of the proposed model is achieved when beam size has the value 6 and the fastest decoding speed of the model is obtained with beam size 6. 

### 6.4. The Effect of the Encoder Self Attention 

The encoding layers’ effectiveness is determined by the capability of several heads of the multi-head attention sub-layer placed inside each layer to capture important structural information. These attention heads, to varying degrees, capture structural information. As remarked by Raganato et al. [48] and Vig et al. [49], some of the heads in multi-head attention sublayer hold the long-distance relations among input token. Other heads in the multi-head attention sub-layer hold the short distance relations among input tokens. This makes the suggested Transformer-Based NMT Subword model obtain the structural and fundamental characteristics efficiently for the input source sentence of Arabic vernacular to increase the effectiveness [48]. As previously stated, the use of Subword units influences how the source language information is handled through the layers of the encoder. As mentioned by Vig et al. [49], this approach is examined through computing two things; the attention entropy and the attention distance spanned through several attention heads within every encoding layer multi-head attention sublayer. The mean distance ${\bar{D}}_{h}^{l}$ that is spanned by attention head h for encoding layer l is calculated a weighted average distance within tokens pairs every sentence of a given corpus X. So:

(19) $${\bar{D}}_{h}^{l}=\frac{\sum_{x\in X}\sum_{i=1}^{\left|x\right|}\sum_{y=1}^{i}w_{i,j}^{h}\cdot \left(i-j\right)}{\sum_{x\in X}\sum_{i=1}^{\left|x\right|}\sum_{y=1}^{i}w_{i,j}^{h}}$$

where $w_{i,j}^{h}$ is attention weight from the input token $x_{i}$ to $x_{j}$ for attention head h.i and *j* signifies the tokens’ places $x_{i}$ and $x_{j}$ in source sentences. By performing aggregation for the attention distance for every head, the mean attention distance spanned ${\bar{D}}^{l}$ with reference to the encoding layer l is computed as:

(20) $${\bar{D}}^{l}=\frac{1}{N_{h}}\cdot \sum_{h=1}^{N_{h}}{\bar{D}}_{h}^{l}$$

where $N_{h}$ indicates the number of attention heads that are used within the layer. Mean attention distance provides no information about how the attention weight is distributed through the input tokens for a particular attention head. Attention head that has greater mean attention distance may concentrate on sequences of same tokens that are separated [49,50]. To estimate the dispersion or concentration pattern for attention head h inside layer l for input token $x_{i}$, entropy of attention distribution [50], $E_{h}^{l}\;\left(x_{i}\right)$ for attention head h is calculated as:

(21) $$E_{h}^{l}\;\left(x_{i}\right)=-\overset{i}{\underset{j=1}{\sum }}w_{i,j}^{h}\log w_{i,j}^{h}$$

The mean entropy of the attention distribution for encoding layer l is computed similarly to the attention distance spanned as:

(22) $$E^{l}\;\left(x_{i}\right)=\frac{1}{N_{h}}\overset{N_{h}}{\underset{h=1}{\sum }}E_{h}^{l}\;\left(x_{i}\right)$$

**Figure 7:**
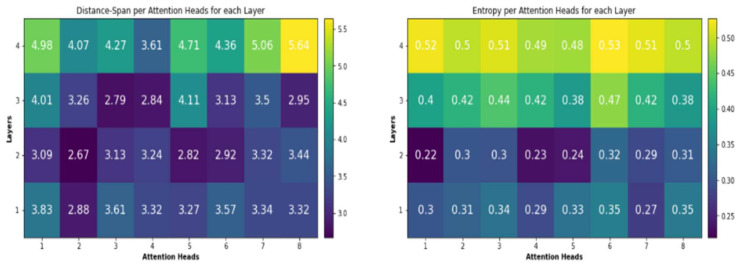
Variation of mean attention distance span and attention distribution entropy with respect to the encoding layers and the attention heads for the suggested model.

**Figure 8:**
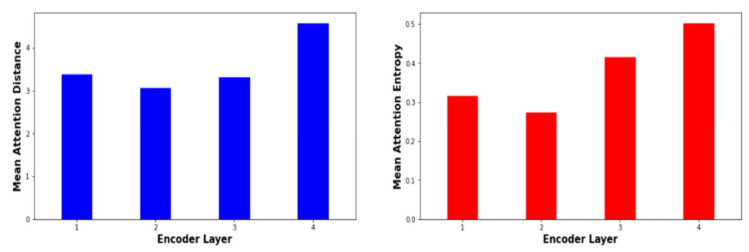
Variation of the average mean attention distance and variation of the average entropy of head attention distribution with respect to each encoder layer for the suggested model.

Attention heads with larger entropy have a more distributed attention pattern, whereas attention heads with a lower entropy have a more focused attention weight distribution. Attention distance and the entropy of attention analysis are conducted based on the attention weights produced for a random 2000 sentences from the MAG–MSA task’s test split (PMM-MAG Corpus). Figure 7 presents mean attention distance span and the mean entropy of attention distribution for every attention head for every encoding layer of the proposed Transformer NMT subword model for Arabic vernaculars. As remarked, some heads focus on short-distance relations among input tokens, other self-attention heads obtain the long-distance relations between input tokens. Furthermore, the entropy of the attention distribution changes through layers. Moreover, the entropy of the attention distribution change for the attention heads within the same layer. The mean average attention distance and entropy for all heads in the multi-head attention through the encoder layers are shown in Figure 8. As shown in Figure 7, for the suggested model, most of the attention heads that have a high mean attention span and much more stable values of attention distribution are located in fourth layer. Despite, a large mean attention distance does not indicate stable attention distribution. The preceding layers have multiple attention heads that have high value of distance span but significantly less consistent attention weights distribution. For instance, in the second layer, attention heads 1 and 4 have the largest mean attention spans (3.09 and 3.24, respectively), but the lowest mean entropy scores (0.22 and 0.23). As Vig et al. [49] highlight, attention heads that have large value of mean attention distance span concentrate their attention to word in repeated sentences that occurs in various places within the source sentence. This can justify their reduced entropy of weight distribution over the sequence of input tokens. Attention heads with a stable or less stable weight distribution and low attention distance span focus significantly more on nearby tokens. Those attention heads that have changeable mean attention distance and changeable entropy enable suggested transformer NMT subword model for Arabic vernaculars to learn efficiently changeable structural information across its layers. This demonstrates the Transformer-Based architecture’s superiority over seq2seq architectures such as RNN and CNN. 

In the proposed model, the Subword units have a strong influence on multi-head attention sub-layer inside the encoding layer. As illustrated in Figure 7 and Figure 8, exposing the encoder layers to the decoder network, enables the encoder subnetwork to learn the source information in a more customized manner. Figure 8 illustrates the change in average mean attention distance span and entropy of the attention weight distribution for different attention heads over various layers of the encoder. As illustrated in Figure 8, the proposed Transformer based subword model concentrated the attention heads that has a shorter attention span over the layers l ≤ 3. These layers are employed to learn the short rang contextual and local knowledge within the neighborhood of input source tokens. The upper layers learn the long-distance interaction within the input source tokens. Generally, utilizing subword units explained how the source information (Arabic dialects) is captured over several attention heads and layers in the encoder as revealed by the entropy of attention weight distribution and attention distance. This improves the proposed Transformer-based NMT subword model’s performance at learning the source semantic information that is required to enhance the quality of translation.

### 6.5. Quantitative Analysis

**Figure 9:**
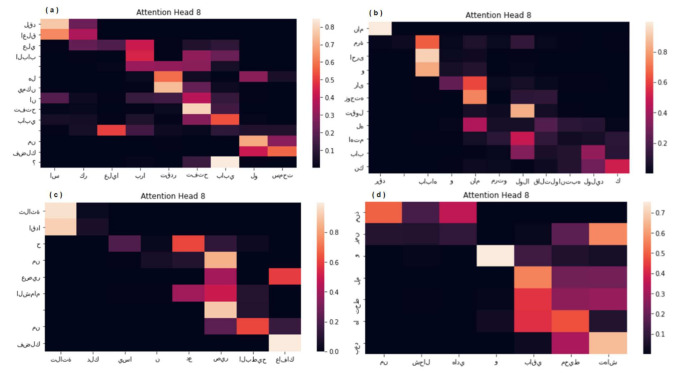
(**a**–**d**) Four Sample Alignments.

The proposed model provides a method of examining the alignment of words in generated translation with words in source sentence. This method is performed through the visualization of annotation weights as illustrated in Figure 9. Every row of the matrix in every plot indicates the weights linked with the annotations where the x-axis represents the input sentence (Maghrebi Arabic) and the y-axis represents the generated sentence in MSA. This reveals which locations in the source sentence were rated more significant when the target word was generated. As illustrated in Figure 9, the alignment of words between Maghrebi Arabic (MAG) and MSA is primarily monotonic. Along the diagonals of each matrix, we see strong weights. Although, we see several non-trivial, non-monotonic alignments. Typically, nouns and adjectives are ordered differently in MAG and MSA, as illustrated in the upper left part of Figure 9. From Figure 9, we see that the model correctly translates a MAG dialect sentence ( اسكر عليا برا تقدر تفتح بابي لو سمحت) “it has been closed from outside, can you open my door please” into MSA sentence (لقد اغلق علي الباب هل يمكن ان تفتح بابي من فضلك ؟) “the door has been closed while I am inside, can you open my door please”. The proposed transformer-based NMT subword model was able to correctly align (اسكر عليا برا) “it has been closed from outside” with (لقد اغلق علي الباب) which means “the door has been closed”, the proposed model was able to understand the contextual clues (الباب) “the door” of Maghrebi Arabic and translated the MAG sentence to MSA correctly. Additionally, the model often handles source and target sentences of variable length. Furthermore, sub-word units approach can be applied on various Arabic NLP tasks such as sentiment analysis [51] and text summarization. 

## 7. Conclusions

This research project introduced a Transformer-Based NMT model for Arabic dialects that utilize Subword units. Through training the suggested model on translation tasks from diverse Arabic dialects to MSA, the model’s translation performance was significantly improved. Utilizing various source representations captured by stacked encoding layers enhance the efficiency of the transformer NMT subword system. The findings of this research project confirm that the Transformer NMT subword model that exploits subword units enhanced the translation BLEU score for MAG–MSA, LEV–MSA, Nile–MSA, Gulf–MSA and IRQ–MSA tasks. The utilization of subword units by using the Wordpiece Model showed that this method is promising and significant for low-resource languages such as Arabic vernaculars. Additionally, using a changeable number of heads in self attention sublayer and training the model with a different number of encoders and decoders improved the quality of translation from Arabic vernaculars to modern standard Arabic. Experimental results on MAG–MSA, LEV–MSA, Nile–MSA, Gulf–MSA and IRQ–MSA translation tasks showed that the proposed model improved the BLEU score’s effectiveness in comparison to other NMT systems. However, the experimental analysis performed reveals that performance gain is reliant on the value of the number of encoding layers considered. Additional analysis reports that increasing the layers of encoder and decoder subnetworks adjust how the local and general contextual knowledge is obtained and captured by employing multi-head attention sublayer used within each encoding layer. The current proposed Transformer-Based NMT subword model can deal with the issue of low availability of Arabic dialects training data. Additionally, the suggested system addressed the Arabic dialect’s grammatical problem; free word ordering. The proposed model with subword units’ utilization is effective and suitable to perform machine translation for low resource languages such as Arabic vernaculars.

## Figures and Tables

**Table 1:** Results of the Transformer-Based NMT Subword Model on PMM-LEV corpus for LEV–MSA translation task, where SW-E-D is the Subword embedding dimension, FS is the filter size, EL is the encoder layer, DL is the decoder layer and AH is the attention heads number.

SW-E-D	FS	EL	DL	AH	BLEU
512	1024	4	4	4	61.65
512	1024	8	8	4	63.56
512	1024	12	12	4	63.71
1024	1024	4	4	4	59.68
1024	1024	4	4	8	59.53
512	512	4	4	4	60.04

**Table 2:** Results of the Transformer-Based NMT Subword Model on PMM–MAG corpus for MAG–MSA translation task, where SW-E-D is the Subword embedding dimension, FS is the filter size, EL is the encoder’s layers, DL is the decoder’s layers and AH is the attention heads number.

SW-E-D	FS	EL	DL	AH	BLEU
512	1024	4	4	4	59.46
512	1024	8	8	4	63.02
512	1024	12	12	4	65.66
1024	1024	4	4	4	59.54
1024	1024	4	4	8	62.17
512	512	4	4	4	56.68

**Table 3:** Results of the Transformer-Based NMT Subword Model on MADAR–Nile Basin corpus for NILE–MSA translation task, where SW-E-D is the Subword embedding dimension, FS is the filter size, EL is the encoder layer, DL is the decoder layer and AH is the attention heads number.

SW-E-D	FS	EL	DL	AH	BLEU
512	1024	4	4	4	47.51
512	1024	8	8	4	48.19
512	1024	12	12	4	47.58
1024	1024	4	4	4	42.02
1024	1024	4	4	8	44.08
512	512	4	4	4	47.52

**Table 4:** Results of the Transformer-Based NMT Subword Model on MADAR–Gulf corpus for GULF–MSA translation task, where SW-E-D is the Subword embedding dimension, FS is the filter size, EL is the encoder layer, DL is the decoder layer and AH is the attention heads number.

SW-E-D	FS	EL	DL	AH	BLEU
512	1024	4	4	4	47.26
512	1024	8	8	4	46.66
512	1024	12	12	4	47.18
1024	1024	4	4	4	43.48
1024	1024	4	4	8	43.68
512	512	4	4	4	46.35

**Table 5:** Results of the Transformer-Based NMT Subword Model on MADAR–Iraqi corpus for IRQ–MSA translation task, where SW-E-D is the Subword embedding dimension, FS is the filter size, EL is the encoder layer, DL is the decoder layer and AH is the attention heads number.

SW-E-D	FS	EL	DL	AH	BLEU
512	1024	4	4	4	56.50
512	1024	8	8	4	49.03
512	1024	12	12	4	47.14
1024	1024	4	4	4	25.51
1024	1024	4	4	8	40.17
512	512	4	4	4	55.23

**Table 6:** Results of the Transformer-Based NMT Subword Model on corpus used by Baniata [22] for MAGHREBI–MSA translation task, where SW-E-D is the Subword embedding dimension, FS is the filter size, EL is the encoder layer, DL is the decoder layer and AH is the attention heads number.

SW-E-D	FS	EL	DL	AH	BLEU
512	1024	4	4	4	57.06
512	1024	8	8	4	57.41
512	1024	12	12	4	57.85
1024	1024	4	4	4	37.15
1024	1024	4	4	8	49.47
512	512	4	4	4	55.14

**Table 7:** Results of the Transformer-Based NMT Subword Model on corpus used by Baniata [22] for LEVANTINE–MSA translation task, where SW-E-D is the Subword embedding dimension, FS is the filter size, EL is the encoder layer, DL is the decoder layer and AH is the attention heads number.

SW-E-D	FS	EL	DL	AH	BLEU
512	1024	4	4	4	56.38
512	1024	8	8	4	53.98
512	1024	12	12	4	57.92
1024	1024	4	4	4	44.13
1024	1024	4	4	8	56.49
512	512	4	4	4	55.46

**Table 8:** Results of Multi-Task NMT Model with POS tagging using FAST Text Embedding that was proposed by Baniata [22].

Model	Pairs	Epochs	Accuracy	BLEU
NMT+POS_LEV	LEV–MSA	90	-	43.00
NMT+POS_LEV	MSA-ENG	50	-	30.00
POS_LEV	POS_LEV	40	98%	-
NMT+POS_MAG	MAG–MSA	50	-	34.00
NMT+POS_MAG	MSA-ENG	30	-	29.00
POS_MAG	POS_MAG	20	99%	-

**Table 9:** Human Evaluation Scores -Pilot Rating Experiments (PRE).

Model	Pairs	Average Score
Transformer-NMT-Subword	LEV–MSA	6.35
Transformer-NMT-Subword	MAG–MSA	6.3
Transformer-NMT-Subword	Gulf–MSA	5.85
Transformer-NMT-Subword	Nile–MSA	5.8
Transformer-NMT-Subword	IRQ–MSA	6.1

**Table 10:** Human Evaluation Scores -PRE for Levantine Arabic (LEV) and Maghrebi Arabic (MAG).

Model	Pairs	Average Score
Transformer-NMT-Subword	LEV–MSA	6.0
Transformer-NMT-Subword	MAG–MSA	6.2
Multi-Task Learning-NMT [22]	LEV–MSA	1.4
Multi-Task Learning-NMT [22]	MAG–MSA	1.3
MTL-NMT+POS [22]	LEV-MAG	5.9
MTL-NMT+POS [22]	MAG–MSA	4.4

**Table 11:** Translation Examples for MAG, LEV, NILE, GULF and IRQ.

Source Language: MAG (Maghrebi)	واخا بلحاق هاد الشي بزاف وانا اصلا واكل بزاف
English Translation (MAG)	Yeah, but that’s so much and I ate lot
Target Language: MSA	اجل ولكن هذا كثير جدا ولقد شبعت بالفعل
Transformer-NMT Subword Model	اجل ولكن هذا كثير جدا ولقد شبعت بالفعل
English translation for output of the Transformer-NMT Subword model	Yes, but that’s too much and I’m already full
Source Language: LEV (Levantine)	انا مع صحابي
English Translation (LEV)	I am with my dudes
Target Language: MSA	انني مع اصدقائي
Transformer-NMT Subword Model	انا مع بعض الاصدقاء
English translation for output of the Transformer-NMT Subword model	I am with some Friends
Source Language: GULF	عندك جوازك وتذكرتك؟ مثل ما تشوف هذي سوق حرة
English Translation (GULF)	You got your passport and your ticket? as you see, this is a duty-free market
Target Language: MSA	هل معك جواز السفر والتذكرة ؟ انت ترى فهذا متجر معفى من الرسوم
Transformer-NMT Subword Model	هل لديك جواز سفرك وتذكرتك ؟ لن يكون عندك متجر معفى من الرسوم
English translation for output of the Transformer-NMT Subword model	Do you have your passport and ticket? You see, this is a duty-free shop
Source Language: Nile (Egypt, Sudan)	عاوز اعمل مكالمة لليابان
English Translation (Nile)	I wanna do a call to Japan
Target Language: MSA	اريد الاتصال هاتفيا باليابان
Transformer-NMT Subword Model	اريد الاتصال هاتفيا باليابان
English translation for output of the Transformer-NMT Subword model	I want to do a phone call to Japan
Source Language: IRQ (Iraqi)	احس ببرودة ومعدتي تاذيني كلش
English Translation (IRQ)	I feel cold and my stomach is hurting me a lot
Target Language: MSA	اشعر ببرودة وتؤلمني معدتي جدا
Transformer-NMT Subword Model	اشعر ببرودة وتؤلمني معدتي جدا
English translation for output of the Transformer-NMT Subword model	I feel cold and my stomach hurts so much

**Table 12:** Translation Examples for Maghrebi Arabic and Levantine Arabic, Baniata [22] Corpus.

Source Language: MAG (Maghrebi)	الى نتي قبلتي تضحي ب هاد الطريقة ف حتى هو خاصو يقدر هاد الامر و ميتخلاش عليك
English Translation (MAG)	If you accept to sacrifice in this way, he is also obliged to appreciate this matter and not abandon you
Target Language: MSA	اذا انت قبلت بان تضحي بهذه الطريقة فهو كذلك مجبر على ان يقدر هذا الامر و الا يتخلى عنك
Transformer-NMT Subword Model	اذا انت قبلت بان تضحي بهذه الطريقة فهو كذلك مجبر على ان يقدر هذا الامر و الا يتخلى عنك
English translation for output of the Transformer-NMT Subword model	If you accept to sacrifice in this way, he is also obliged to appreciate this matter and not abandon you
Source Language: LEV (Levantine)	اه ريحتها زي ريحة العطر منيحة في اليوم الاول بس بعد هيك
English Translation (LEV)	yeah, it smells like perfume, good on the first day, but later on
Target Language: MSA	نعم رائحتها كرائحة العطر جيدة في اليوم الاول لكن فيما بعد
Transformer-NMT Subword Model	نعم رائحتها كرائحة العطر جيدة في اليوم الاول لكن فيما بعد
English translation for output of the Transformer-NMT Subword model	Yes, it smells as good as perfume on the first day, but later on

**Table 13:** Change in BLEU Score According to Beam Size.

Beam Size	BLEU
1	52.82
2	58.10
3	59.69
4	60.31
5	61.70
6	65.66
7	61.22
8	61.35
9	60.72
10	61.36

## Data Availability

The datasets generated during the current study are available in [Transformer_NMT_AD] repository (https://github.com/laith85/ (accessed on 26 September 2021). Transformer_NMT_AD).
